# Impact of intrauterine exposure to maternal diabetes on preterm birth: fetal DNA methylation alteration is an important mediator

**DOI:** 10.1186/s13148-023-01473-1

**Published:** 2023-04-07

**Authors:** Guoying Wang, Richard Xu, Boyang Zhang, Xiumei Hong, Tami R. Bartell, Colleen Pearson, Liming Liang, Xiaobin Wang

**Affiliations:** 1 Center on Early Life Origins of Disease, Department of Population, Family and Reproductive Health, Johns Hopkins University Bloomberg School of Public Health, 615 N. Wolfe Street, Baltimore, MD 21205-2179 USA; 2 Department of Biostatistics, Johns Hopkins University Bloomberg School of Public Health, Baltimore, MD USA; 3 Patrick M. Magoon Institute for Healthy Communities, Ann & Robert H. Lurie Children’s Hospital of Chicago, Chicago, IL USA; 4 Department of Pediatrics, Boston University Chobanian & Avedisian School of Medicine and Boston Medical Center, Boston, MA USA; 5 Department of Epidemiology, Harvard T.H. Chan School of Public Health, Boston, MA USA; 6 Department of Biostatistics, Harvard T.H. Chan School of Public Health, Boston, MA USA; 7 Department of Pediatrics, Johns Hopkins University School of Medicine, Baltimore, MD USA

**Keywords:** Cord blood, Diabetes, DNA methylation, In utero, Preterm birth

## Abstract

**Background:**

In utero exposure to diabetes has been shown to contribute to preterm birth, though the underlying biological mechanisms are yet to be fully elucidated. Fetal epigenetic variations established in utero may be a possible pathway. This study aimed to investigate whether in utero exposure to diabetes was associated with a change in newborn DNA methylation, and whether the identified CpG sites mediate the association between diabetes and preterm birth in a racially diverse birth cohort population.

**Methods:**

This study included 954 mother–newborn pairs. Methylation levels in the cord blood were determined using the Illumina Infinium MethylationEPIC BeadChip 850 K array platform. In utero exposure to diabetes was defined by the presence of maternal pregestational or gestational diabetes. Preterm birth was defined as gestational age at birth less than 37 weeks. Linear regression analysis was employed to identify differentially methylated CpG sites. Differentially methylated regions were identified using the DMRcate Package.

**Results:**

126 (13%) newborns were born to mothers with diabetes in pregnancy and 173 (18%) newborns were born preterm, while 41 newborns were born both preterm and to mothers with diabetes in pregnancy. Genomic-wide CpG analysis found that eighteen CpG sites in cord blood were differentially methylated by maternal diabetes status at an FDR threshold of 5%. These significant CpG sites were mapped to 12 known genes, one of which was annotated to gene Major Histocompatibility Complex, Class II, DM Beta (HLA-DMB). Consistently, one of the two identified significant methylated regions overlapped with HLA-DMB. The identified differentially methylated CpG sites mediated the association between diabetes in pregnancy and preterm birth by 61%.

**Conclusions:**

In this US birth cohort, we found that maternal diabetes was associated with altered fetal DNA methylation patterns, which substantially explained the link between diabetes and preterm birth.

**Supplementary Information:**

The online version contains supplementary material available at 10.1186/s13148-023-01473-1.

## Background

With the increasing prevalence of obesity, the rates of type 2 diabetes and gestational diabetes (GDM) have been rising globally [1]. The consequences of these rising rates are that in utero exposure to diabetes has become a common scenario that is linked to a wide range of detrimental health outcomes [2–4]. A growing number of studies have demonstrated that in utero exposure to diabetes has contributed to adverse birth outcomes, such as preterm birth and macrosomia [3, 4]. Along the same lines, even maternal hyperglycemia which did not met the criteria of diabetes diagnosis was also significantly associated with preterm birth [5]. However, the underlying biological mechanisms driving these outcomes are yet to be fully elucidated.

Preterm birth, a birth occurring at less than 37 completed gestational weeks, has been identified as the most important cause of perinatal mortality and infant morbidity and has a lifelong impact [6]. Despite limited understanding about the root cause and biological mechanisms underlying preterm birth, it is increasingly recognized that genetic and environmental interaction plays a critical role [7, 8], while epigenetic mechanisms are likely the interface that coordinates environmental factors with genes to drive such changes [9]. Studies have provided evidence that fetal epigenetic variations [10], which are largely established in utero [11], may contribute to preterm birth [12]. Several studies have documented that maternal diabetes in pregnancy was associated with altered methylation levels in cord blood DNA [13–15]. However, little is known about whether and to what degree the influence of diabetes has on preterm birth through variation in DNA methylation.

Using the Boston Birth Cohort (BBC), a well-established racially and ethnically diverse birth cohort, we sought to identify the associations between exposure to diabetes in utero (assessed as maternal diabetes in pregnancy) and DNA methylation changes in cord blood. Next, we further explored whether these differentially methylated positions (DMPs) mediated the association between diabetes and preterm birth.

## Results

### Study characteristics

This study included 954 maternal-infant dyads. Mean (standard deviation (SD)) maternal age at delivery was 28.3 (6.6) years. Among the mothers, 679 (71.2%) self-identified as non-Hispanic Black and 126 (13.2%) experienced diabetes (including GDM (*n* = 83), type 2 diabetes (*n* = 39), and type 1 diabetes (*n* = 4)). Compared to mothers with GDM, mothers who had pregestational diabetes had higher blood glucose concentrations and a higher proportion used insulin as treatment (Additional file 1: Table S1). Among the newborns, 504 (52.8%) were male and 173 (18.1%) were born prematurely, of which 41 newborns were born to mothers with diabetes in pregnancy. Maternal and neonatal characteristics are shown in Table 1. As expected, mothers with diabetes were more likely to be older, had higher prepregnancy body mass index (BMI), and were more likely to be with overweight or obesity. Newborns of mothers with diabetes were more likely to have shorter gestation and to be born prematurely, to be large for gestational age (LGA), and to have lower proportions of granulocytes and nature killer cells and higher proportion of nucleated red blood cells in their cord blood. An overall predominance of non-Hispanic Black race and ethnicity was observed among participating mothers both with and without diabetes.

**Table 1:** Characteristics of maternal-infant pairs

	Maternal diabetes status		
Characteristics	Non-diabetes	Diabetes	*P* value
*n*	828	126	
Maternal characteristics			
Maternal age (years)	28.0 ± 6.5	30.8 ± 6.3	< 0.001
Race (non-Hispanic Black)	588 (71.0)	91 (72.2)	0.862
Education (college and above)	282 (34.1)	38 (30.2)	0.446
Parity (multiparous)	442 (53.4)	84 (66.7)	0.007
Smoking	142 (17.1)	23 (18.3)	0.858
Body mass index (kg/m^2^)	26.3 ± 5.9	30.8 ± 8.1	< 0.001
Overweight or obesity at prepregnancy	409 (49.4)	97 (77.0)	< 0.001
Neonatal characteristics			
Sex (male)	428 (51.7)	76 (60.3)	0.087
Birthweight (g)	3,134 ± 631	3,059 ± 870	0.241
Gestational age (wks)	38.8 ± 2.3	37.3 ± 3.4	< 0.001
Preterm birth	132 (15.9)	41 (32.5)	< 0.001
Subtype of preterm birth			0.063
Spontaneous preterm	102(77.3)	25(61.0)	
Medically indicated preterm	30(22.7)	16(39.0)	
Low birthweight	131 (15.8)	27 (21.4)	0.147
Fetal growth			< 0.001
Appropriate for gestational age	664 (80.2)	90 (71.4)	
Small for gestational age	90 (10.9)	7 (5.6)	
Large for gestational age	74 (8.9)	29 (23.0)	
Predicted cord blood cell proportions			
CD8 T cells	0.09 ± 0.03	0.09 ± 0.03	0.589
CD4 T cells	0.12 ± 0.05	0.12 ± 0.05	0.899
Natural killer cells	0.06 ± 0.03	0.05 ± 0.03	0.036
B cells	0.08 ± 0.03	0.09 ± 0.04	0.108
Monocytes	0.12 ± 0.03	0.11 ± 0.03	0.650
Granulocytes	0.37 ± 0.14	0.31 ± 0.15	< 0.001
Nucleated red blood cells	0.18 ± 0.14	0.25 ± 0.18	< 0.001

### Epigenome-wide analysis of differentially methylated positions (DMPs)

Quantile–quantile (Q-Q) plots and lambda (λ) values (1.14) are provided in Additional file 1: Fig. S1. Epigenome-wide associations between diabetes in pregnancy and cord blood DNA methylation are visualized in the Manhattan plot (Additional file 1: Fig. S2). As shown in Table 2, 18 CpG probes were identified as significantly differentially methylated at a false discovery rate (FDR) of *P* < 0.05 and annotated to 12 known genes. Among these DMPs, the top DMP was located within the 5 prime untranslated region (5’UTR) of the Leucine Rich Repeat Containing 8 VRAC Subunit B (LRRC8B) gene, which is involved in anion transmembrane transport; two top DMPs were located in genes Dynamin 3 (DNM3) and Family With Sequence Similarity 163 Member A (FAM163A), which are involved in components and functions of membranes; and another top DMP was mapped to the body of gene Major Histocompatibility Complex (MHC), Class II, DM Beta (HLA-DMB), which is associated with autoimmune diseases. Of these DMPs, 17 (94.4%) showed hypermethylation in newborns whose mothers had diabetes in pregnancy, whereas only 1 DMP (cg21363811) located within the CpG island of the FAM163A gene showed hypomethylation (Table 2). When analyses were conducted separately by GDM and pregestational diabetes, the association patterns were similar (Additional file 1: Table S2). Furthermore, no significantly differential methylation levels were found in the identified DMPs between GDM and pregestational diabetes (Additional file 1: Table S3).

**Table 2:** CpGs in cord blood associated with intrauterine diabetes exposure

Cpg ID	Coef	SD	*P* value	Adj P	CH	Coordinate	Target Gene	Genomic location	Relation to CpG Island
cg19032863	0.464	0.085	5.83E−08	0.017	1	90011484	LRRC8B	5'UTR	
cg18997837	0.365	0.066	6.06E−08	0.017	1	171882657	DNM3	Body	
cg08810410	0.327	0.060	9.22E−08	0.017	1	203300522			S_Shelf
cg21363811	− 0.450	0.088	3.53E−07	0.031	1	179713950	FAM163A	5'UTR	Island
cg24804643	0.433	0.089	1.15E−06	0.045	1	197455252			
cg15317464	0.430	0.085	4.65E−07	0.034	2	9105414	MBOAT2	Body	
cg25049210	0.438	0.086	3.71E−07	0.031	3	30495126			
cg22324029	0.473	0.092	3.31E−07	0.031	6	32908466	HLA-DMB	Body	
cg08840298	0.248	0.049	8.32E−07	0.044	9	124044927	GSN	5'UTR Body	
cg21747782	0.291	0.057	6.88E−07	0.041	10	126103031	OAT	5'UTR	N_Shelf
cg25953130	0.458	0.094	1.24E−06	0.045	10	63753550	ARID5B	Body	
cg09191149	0.460	0.093	8.70E−07	0.044	16	10962161			
cg00938688	0.381	0.077	9.34E−07	0.044	16	11862556	ZC3H7A	Body	
cg04384031	0.340	0.063	1.03E−07	0.017	17	19631485			S_Shelf
cg04492567	0.326	0.062	2.35E−07	0.031	17	19631464			S_Shelf
cg07408552	0.426	0.087	1.00E−06	0.044	17	40611995	ATP6V0A1	5'UTR	S_Shore
cg09915396	0.345	0.070	1.07E−06	0.044	17	2907895	RAP1GAP2	Body	
cg18575710	0.431	0.085	5.30E−07	0.035	22	28569035	TTC28	Body	

*Adj P* P value adjusted by multiple test correction, *CH* Chromosome, *Coef* Coefficient

Models were adjusted for maternal age, race and ethnicity, educational attainment, smoking status during pregnancy, prepregnancy overweight or obesity, newborn’s sex, fetal growth status, and cell type composition

### Identification of differentially methylated regions (DMRs)

DMRs were investigated using ‘DMRcate’ by windows 500- and 1000-bp. Of the 4 DMRs identified by window 500-bp, 2 were significant using an FDR threshold of 0.10, including chromosome 17: 19631464–19631485, which contained 2 probes and lacked overlapping known genes and chromosome 6: 32908466–32908718, which contained 5 probes located within the 5’UTR and first exon as well as the body of the HLA-DMB gene, which is associated with autoimmune diseases [16–18] (Table 3). All probes covered in the two significant DMRs had higher methylation levels in the cord blood of mothers with diabetes (Additional file 1: Table S4). Although three DMRs were identified by window 1000-bp, none of them survived after correction for multiple testing (Additional file 1: Table S5).

**Table 3:** Differentially methylated regions (DMRs) identified by DMRcate in 500 bp window

DMR coordinates	Chr	CpGs comprising the DMR	Width	No. of CpG	Overlapping genes	Fisher	HMFDR
19631464–19631485	17	cg04384031; cg04492567	22	2		0.004	0.022
32908466–32908718	6	cg00575744; cg10310595; cg12840248; cg16236263; cg22324029	253	5	HLA-DMB	0.030	0.089
57915665–57915740	17	cg12054453; cg01409343; cg16936953	76	3	VMP1	0.103	0.151
32120863– 32120955	6	cg13934406; cg06025456; cg23359665; cg02956248; cg17113856; cg16481280; cg06108383	93	7	PRRT1	0.923	0.588

*Chr* Chromosome, *Fisher* Fisher's multiple comparison statistic, *HMFDR* Harmonic mean of the individual component FDRs

### Lookups in the literature

Of 10 studies [13–15, 19–25] measured DNA methylation using the 450 K or 850 K microarray and investigated the associations between maternal pregestational diabetes/GDM and cord blood DNA methylation, 4 studies [13, 14, 22, 23] reported DMPs on EWAS. Two study was removed due to small sample size of GDM cases (*n* < 50) [22, 23]. Across the 2 studies [13, 14], 307 unique CpGs were associated with diabetes exposure after adjustment for multiple comparisons. Of these CpGs, 274 were evaluated in the current study. Of 274 CpGs examined, 120 were nominally associated with diabetes in pregnancy and 115 with the same direction of effect, of which, 87 CpGs were significant with an FDR-corrected P value less than 0.05 based on 274 tests conducted (Additional file 1: Table S6).

### Gene set enrichment analysis

We did not find statistically significant enriched pathways using ontologies defined in the KEGG (Kyoto Encyclopedia of Genes and Genomes) and GO (Gene Ontology) databases at an FDR *p* < 0.05. However, as shown by the enrichment results at both the DMP-level and DMR-level, the top 10 KEGG pathways showed enrichment of inflammatory and immune-related pathways (Additional file 1: Tables S7 and S9). Notably, the top 10 KEGG pathways in DMRs identified by windows of both 500-bp and 1000-bp were highly concordant. Along this same functional theme, GO terms for DMRs showed enrichment of immune response (Additional file 1: Tables S10), while GO terms for DMPs included multiple arginine metabolism pathways, which are involved in cellular metabolism and function [26] (Additional file 1: Table S8).

### Mediation effect of DMPs

The associations of diabetes in pregnancy and 18 identified DMPs with preterm birth and subtypes are presented in Additional file 1: Table S11. Diabetes in pregnancy and 17 DMPs were significantly associated with an increased risk of preterm birth, especially medically indicated preterm birth. One CpG (cg21363811) mapped to gene FAM163A was associated with a reduced risk of preterm birth. Among the 18 DMPs, 5 were selected as mediators in the association between diabetes in pregnancy and preterm birth at a *p* level of < 0.10 (Additional file 1: Table S12). Taken as a whole, the 5 DMPs mediated 61% of the association (Table 4). In sensitivity analyses, we evaluated the mediation effect of these DMPs on medically indicated preterm. About 66% of the associations of diabetes with indicated preterm birth were explained by these DMPs (Additional file 1: Table S13).

**Table 4:** Mediation effect of CpGs on the association between diabetes in pregnancy and preterm birth

Mediator	TE/DE (95%CI)	IE (95%CI)	RE (%)
Total effect	1.910(1.094, 2.845)		
Direct effect	0.516(− 0.125, 1.105)		0.270(− 0.072. 0.536)
cg08810410		− 0.131(− 0.353, 0.071)	− 0.068(− 0.198, 0.043)
cg25953130		0.272(0.039, 0.476)	0.143(0.026, 0.240)
cg09191149		0.224(0.076, 0.405)	0.117(0.029, 0.230)
cg09915396		0.349(0.142, 0.609)	0.183(0.068, 0.329)
cg07408552		0.440(0.191, 0.773)	0.230(0.100, 0.408)
Joint effect of CpGs		1.169(0.714, 1.722)	0.612(0.380, 0.900)

*DE* Direct effect, *IE* Indirect effect, *RE* Relative effect, *TE* Total effect

The model was adjusted for maternal age at delivery, race and ethnicity, educational attainment, smoking status during pregnancy, prepregnancy overweight or obesity, neonatal sex, and neonatal birthweight. The response variable is on the scale of log-odds of being preterm. Significance level was set at 0.1

## Discussion

In this study, we examined the associations between maternal diabetes in pregnancy and cord blood DNA methylation patterns at both individual and regional CpG levels. Eighteen individual CpG sites and two regions were identified to differentially methylate by diabetes in pregnancy. In our data, the directions of the effect for DMPs were mostly positive, indicating a hypermethylation effect. Furthermore, our data showed that diabetes in pregnancy was associated with an increased risk of preterm birth, and about 61% of the association was explained by the DMPs.

LRRC8B, encoding leucine-rich repeat containing 8 family, member B, is one of the LRRC8 gene family members and LRRC8B protein acting as a leak channel in the endoplasmic reticulum plays an important role in intracellular Ca^2+^ homeostasis [27]. Although altered methylation in gene LRRC8B has not been previously reported in existing literature as differentially methylated in cord blood in regard to maternal diabetes in pregnancy, cell-type-specific methylation changes in gene of LRRC8B have been associated with Alzheimer's disease [28]. Previous studies have also implicated the role of prenatal exposure to diabetes in autism and neuropsychiatric disease [29, 30]. Another hit was on cg25953130, located in the body of ARID5B gene which is involved in the regulation of the transcription of target genes involved in adipogenesis and liver development and plays a role in cell growth and differentiation of B-lymphocyte progenitors [31]. In agreement with our findings, Antoun et al. also found that a CpG site which was annotated to the ARID5B gene was positively associated with GDM [13].

One DMP (cg22324029) was annotated to the HLA-DMB gene, which was previously linked to several autoimmune diseases, such as multiple sclerosis [16], rheumatoid arthritis [17], and type 1 diabetes [18]. Consistently, one significant DMR (Chr 6, 32908239–32909282) also overlapped with the HLA-DMB gene, suggesting that the association between diabetes in pregnancy and the CpG site was robust. More importantly, this DMR covered 4 CpG sites in the 5’UTR and exon 1 of HLA-DMB, indicating the regulatory functions of the DMR. HLA-DMB belongs to the HLA class II beta chain paralogs, which play a critical role in the peptide loading of major histocompatibility complex (MHC) class II molecules. The abnormal DNA methylation of HLA-DMB modified the gene expression and was associated with an increased risk of asthma [32], suggesting HLA-DMB is involved in human immune response. In support of our finding, a previous study reported an association between GDM and a CpG site located in gene MHC, class II, DR beta (HLA-DRB) [13], an important paralog of HLA-DMB; and alterations in DNAm of the MHC region in placentas from mothers with GDM [25, 33], as well as in cord blood of offspring from mothers with GDM [24]. In addition, strong enrichment of inflammation and immune response pathways of DNAm was also found in human amniocytes exposed to GDM in utero [34]. Gene set enrichment analysis also showed that these identified DMPs and DMRs were involved in the pathways that are integral to autoimmune diseases. Previous studies showed that maternal hyperglycemia can exacerbate inflammatory and immune responses during pregnancy, leading to adverse fetal outcomes [35]. Epidemiological study also provides evidence that GDM increases the risk of asthma in the offspring [36].

In agreement with previous studies which reported a positive association between preterm birth and type 1 diabetes [3], type2 diabetes [4], and GDM [37], our data showed a strong association between diabetes in pregnancy and preterm birth and its subtype (medically indicated preterm birth). We explored beyond previous studies and demonstrated that the DMPs induced by diabetes mediated the association by more than two-thirds. Since diabetes-induced DMPs were enriched in the immune response and inflammatory pathway, our data support the hypothesis implicating the involvement of inflammatory and immune response in the etiology of preterm birth [38]. In further support of our findings, one study using two dependent cohorts also showed that the preterm birth-associated CpGs were mapped to genes enriched for inflammation and immune pathways [12].

This study has several strengths. First, to the best of our knowledge, this study is the first to explore the mediation effect of DNA methylation patterns induced by diabetes in pregnancy on the associations between diabetes and preterm birth. Second, our study examined the DMRs in addition to DMPs.

Several limitations should also be acknowledged. First, the number of newborns born to mothers with diabetes in pregnancy included in this study was relatively small, especially for that coupled with preterm birth. Second, a lambda of 1.14 suggests that not all possible test-statistic inflation has been ruled out. Third, due to the combination of GDM with pregestational diabetes, we cannot differentiate the effects of the two diabetic conditions on the cord blood methylation. Unavailability of blood glucose data for mothers without diabetes limited our ability to investigate the effect of glycemia on the methylation. Fourth, although the look-up analysis showed that some DMPs identified in our study were consistent with previous studies, future studies are needed to replicate our results in larger samples. Fifth, because of a substantial missing in the genotyping data, we could not perform mQTL analysis. Finally, the study population is a predominantly urban and primarily Black cohort from low-resourced communities and households; thus, caution is needed when assessing the generalizability of the findings to other populations with different characteristics.

## Conclusion

This study found that exposure to diabetes in utero was associated with DMPs and DMRs. Furthermore, these identified DNA methylation alterations substantially mediated the association between diabetes in pregnancy and preterm births.

## Materials and methods

### Study design and population

The study population was a subset of the BBC, an ongoing prospective birth cohort, recruited at 2–3 days after delivery at Boston Medical Center, MA, USA, starting in 1998. A detailed description of the BBC has been reported previously [39]. In brief, at enrollment, each mother completed a questionnaire interview that gathered demographic information. Cord blood was collected immediately after birth. As of December 2018, 8623 mother-infant pairs had been enrolled. Of these pairs, 963 cord blood samples were analyzed by DNA methylation array. Of these, 9 samples were removed due to not passing the quality control process. Finally, this study included 954 mother-infant pairs. Maternal demographic characteristics and birth outcomes were comparable between the study sample included and the total BBC, except that more Black participants and preterm births among the sample were included in this study (Additional file 1: Table S14). The study protocol was approved by the institutional review boards of Boston University Medical Center and Johns Hopkins Bloomberg School of Public Health. Written informed consent was obtained from all the study mothers.

### Definition of maternal diabetes status in pregnancy

Diabetes in pregnancy included pregestational diabetes (type 1 and type 2 diabetes) and GDM, which was abstracted from electronic medical records (EMRs) and verified by blood glucose profiles extracted from EMRs. Pregestational diabetes was defined as fasting glucose concentration ≥ 7 mmol/L, 2 h plasma glucose of 75 g oral glucose tolerance test (OGTT) or random plasma glucose ≥ 11.1 mmol/L, HbA1c ≥ 6.5%, or receiving treatment for diabetes prior to pregnancy or in the first trimester [40]. GDM was diagnosed if women were free from pregestational diabetes and at least two of the following plasma glucose values were met: fasting glucose ≥ 5.3 mmol/l, 1 h ≥ 10.0 mmol/l, 2 h ≥ 8.6 mmol/l, and 3 h ≥ 7.8 mmol/l in response to a 100 g oral glucose load around 20–32 weeks of the index pregnancy according to American Diabetes Association criteria [40].

### Definition of preterm birth

Gestational age was abstracted from EMRs. Preterm birth was defined as gestational age < 37 weeks and then further classified into two subtypes: spontaneous preterm birth (spontaneous preterm labor, preterm prelabor rupture of membranes, or cervical insufficiency) and medically indicated preterm birth (indicated by a specific maternal or fetal complication) according to the definition of the American College of Obstetricians and Gynecologists [41].

### Maternal and neonatal characteristics

Maternal socio-demographic information was collected using a questionnaire interview. Educational attainment was classified into high school and below vs college and above; maternal smoking during pregnancy was classified into never smoker vs smoker; and maternal race and ethnicity were classified as non-Hispanic Black vs. non-Black (which included non-Hispanic White, Hispanic, Asian, Pacific Islander, and mixed race) based on self-reported race and ethnicity. Prepregnancy BMI was calculated as prepregnancy weight (kg) divided by height (m) squared. Maternal overweight and obesity were defined as BMI ≥ 25 kg/m^2^ [42]. Neonatal sex and birth weight were abstracted from EMRs. Fetal growth was assessed by birthweight for gestational age and categorized into 3 groups: small for gestational age (SGA, < 10th percentile), LGA (> 90th percentile), and appropriate for gestational age (AGA, 10th-90th percentile) according to an established local sex and race-specific reference population [43].

### Genome-wide DNA methylation assessment in cord blood

Cord blood samples were collected at birth for genomic DNA extraction. DNA methylation profiling was analyzed using the Illumina Infinium MethylationEPIC BeadChip 850 K array platform (Illumina, California, USA) at the University of Minnesota Genomics Center, Minneapolis, MN, USA. Systematic quality control steps were reported in a previous publication [44]. In brief, profiling was conducted using existing analytic pipelines with R/Bioconductor package minfi [45]. At the sample level, we removed samples that appeared to be outliers (*n* = 1) or had a high missing rate (*n* = 1) or mismatch between data abstracted from EMRs and predicted sex (*n* = 7). At the probe level, we excluded probes with detection P values > 0.01 (*n* = 4,193), probes with known single nucleotide polymorphisms within the CpG motif or that have been shown to cross-hybridize (*n* = 140,271), probes with missing values (*n* = 44,900), and probes located on the X and Y chromosomes (*n* = 14,499). After these exclusions, a total of 661,996 CpG sites were interrogated in this study.

### Statistical analysis

Socio-demographic and clinical variables are presented as mean ± SD for continuous variables and frequencies with proportions for categorical variables across maternal diabetes status in pregnancy. Raw methylation (beta) values were converted to M-values to better approximate a normal distribution.

Linear regression analysis was performed using the ‘limma’ package [46] to examine the association of maternal diabetes in pregnancy with each DNA CpG site in cord blood. Maternal diabetes in pregnancy was modeled as the exposure of interest, and cord blood DNA methylation levels (M-values) at each CpG site were modeled as the response variable, adjusted for maternal age, educational attainment, race and ethnicity, smoking during pregnancy, prepregnancy overweight or obesity, infant’s sex, fetal growth, and cell proportions (B cells, CD8 T cells, CD4 T cells, granulocytes, natural killer cells, monocytes, and nucleated red blood cells) which were estimated based on cord blood reference data [47] using ‘minfi’ package, as reported previously [44]. Genomic inflation was evaluated by constructing Q-Q plots and calculating λ for the epigenome-wide analyses. We corrected for multiple testing using the Benjamini–Hochberg FDR procedure. DMRs that were associated with maternal diabetes were identified using the Bioconductor package DMRcate [48] with the default settings using the same model and covariates as the abovementioned DMPs analysis.

A logistic regression model was applied to examine the association between maternal diabetes in pregnancy and preterm birth. To examine whether the identified DMPs mediated the association between diabetes and preterm birth, we performed mediation analysis for multiple mediators simultaneously using mma package [49], which allows for correlations among mediators. To evaluate the potential biological pathways in which diabetes-associated DMPs or DMRs were implicated, we performed probe-wise and region-based gene set enrichment analysis using GOmeth and GOregion functions in missMethyl R package [50], respectively. All statistical analyses were performed using the R statistical package, version 4.1.2 (R Foundation for Statistical Computing, Vienna).

## Supplementary Information

**Additional file 1**. Supplementary figures and tables.

## Data Availability

The datasets supporting these findings are not publicly available. The datasets used and/or analyzed for the current study are available from the corresponding author on reasonable request and after Institutional Review Board review and approval.

## Abbreviations

AGA: Appropriate for gestational age
BBC: Boston Birth Cohort
BMI: Body mass index
DE: Direct effect
DMP: Differentially methylated position
DMR: Differentially methylated region
EMR: Electronic medical record
GDM: Gestational diabetes mellitus
GO: Gene Ontology
IE: Indirect effect
KEGG: Kyoto Encyclopedia of Genes and Genomes
LGA: Large for gestational age
OGTT: Oral glucose tolerance test
RE: Relative effect
SGA: Small for gestational age
TE: Total effect
