# Supplementary material for: Impact of intrauterine exposure to maternal diabetes on preterm birth: fetal DNA methylation alteration is an important mediator
Source: Clin Epigenetics. 2023 Apr 7;15:59. doi: 10.1186/s13148-023-01473-1 (PMC10082529; doi:10.1186/s13148-023-01473-1)
Supplement: Supplementary file 1 — Additional file 1. Supplementary figures and tables. [file 13148_2023_1473_MOESM1_ESM.pdf]

Supplementary Figure S1. Q-Q plots of associations between maternal diabetes in pregnancy and epigenome-wide cord blood DNA methylation.

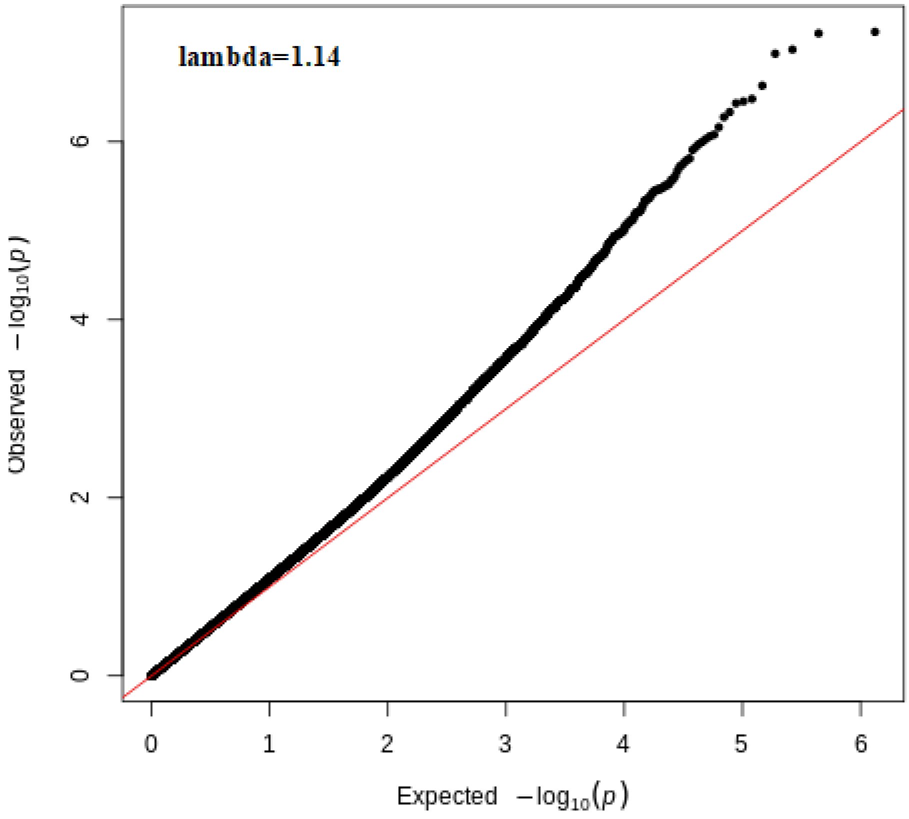

Supplemental Figure S2. Manhattan plot of associations between maternal diabetes and epigenome-wide cord blood DNA methylation.

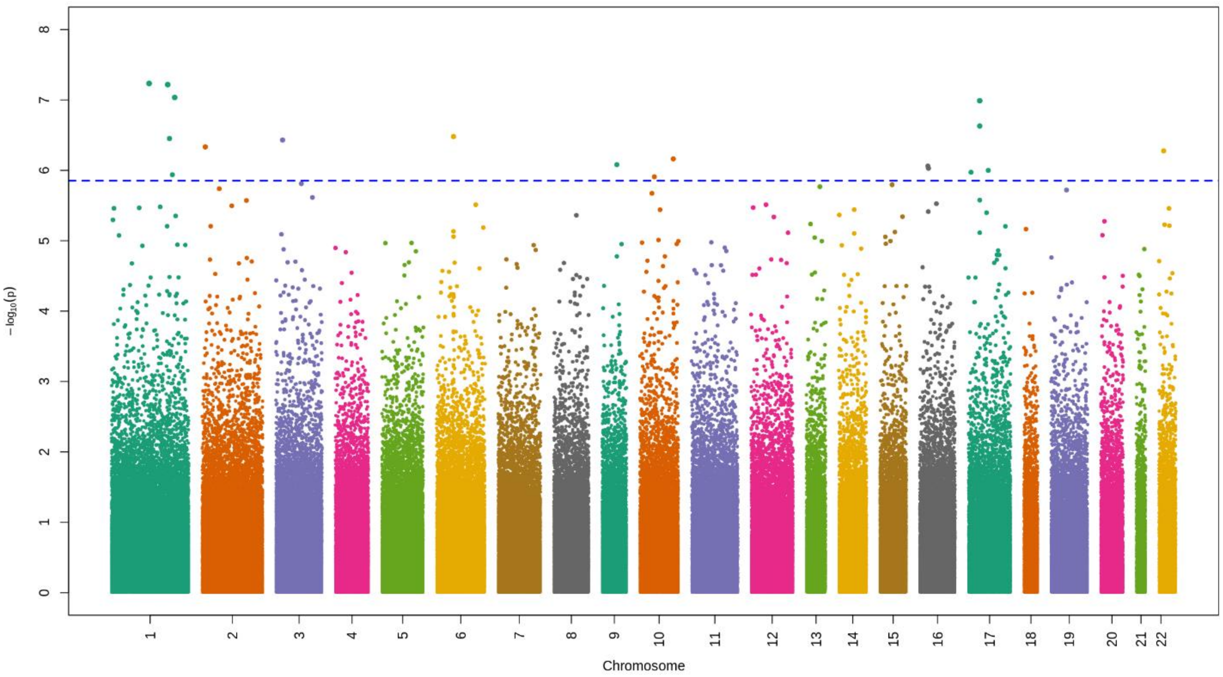

Dashed line represents FDR corrected p value threshold for significance (<0.05).

Supplemental Table S1. Mean blood glucose levels and treatment during pregnancy in mothers with diabetes in pregnancy

Blood glucose or treatment	Total diabetes		Gestational diabetes		Pregestational diabetes		
	n	Mean±SD	n	Mean±SD	n	Mean±SD	P value
**Blood glucose**							
Fasting	42	90.0±13.0	39	89.3±13.4	3	104.2±11.5	0.069
Random	95	103.3±34.7	59	99.4±29.9	36	109.6±41.2	0.204
Point of care	108	99.7±26.0	71	94.9±25.2	37	109.0±25.2	0.007
**Treatment**							
Insulin	42	-	14	-	28	-	-
Sulfonylureas	13	-	11	-	2	-	-

Supplemental Table S2. Associations of gestational diabetes and pregestational diabetes with identified CpGs

	Gestational diabetes				Pregestational diabetes			
CpG ID	β	se	p1	p_fdr	β	se	p	p_fdr
cg19032863	0.016	0.005	1.2E-03	1.2E-03	0.029	0.006	3.5E-06	4.5E-05
cg18997837	0.051	0.010	4.5E-07	4.1E-06	0.044	0.013	6.5E-04	1.1E-03
cg21363811	-0.037	0.008	4.5E-06	1.8E-05	-0.033	0.011	2.4E-03	3.1E-03
cg24804643	0.017	0.005	2.7E-04	3.3E-04	0.019	0.006	2.6E-03	3.1E-03
cg08810410	0.027	0.006	5.1E-06	1.8E-05	0.029	0.008	2.2E-04	6.8E-04
cg15317464	0.037	0.008	1.5E-05	3.8E-05	0.039	0.011	6.0E-04	1.1E-03
cg25049210	0.030	0.009	1.0E-03	1.2E-03	0.036	0.012	3.6E-03	4.1E-03
cg22324029	0.054	0.010	1.0E-07	1.8E-06	0.047	0.013	4.0E-04	9.0E-04
cg08840298	0.019	0.005	6.1E-05	9.9E-05	0.028	0.006	9.4E-06	5.7E-05
cg25953130	0.034	0.011	2.3E-03	2.3E-03	0.068	0.015	5.0E-06	4.5E-05
cg21747782	0.019	0.005	1.1E-04	1.4E-04	0.019	0.007	5.4E-03	5.7E-03
cg09191149	0.031	0.007	4.5E-05	8.1E-05	0.037	0.010	2.3E-04	6.8E-04
cg00938688	0.025	0.006	8.8E-05	1.2E-04	0.032	0.009	2.8E-04	7.2E-04
cg09915396	0.028	0.006	1.6E-06	9.6E-06	0.017	0.007	1.8E-02	1.8E-02
cg04492567	0.019	0.005	3.1E-05	6.3E-05	0.021	0.006	7.2E-04	1.1E-03
cg04384031	0.019	0.004	1.8E-05	4.1E-05	0.022	0.006	8.6E-05	3.9E-04
cg07408552	0.021	0.005	1.4E-05	3.8E-05	0.021	0.006	9.9E-04	1.4E-03
cg18575710	0.024	0.006	8.9E-05	1.2E-04	0.029	0.008	4.7E-04	9.4E-04

Models were adjusted for maternal age, race and ethnicity, education attainment, smoking status during pregnancy, prepregnancy overweight or obesity, newborn's sex, fetal growth status, and cell type composition.

Supplemental Table S3. Comparison of cord blood DNA methylation levels between newborns born to mothers with GDM and those born to mothers with pregestational diabetes

CpG ID	beta	se	P value
cg19032863	0.003	0.011	0.794
cg18997837	-0.007	0.023	0.749
cg21363811	0.003	0.015	0.858
cg24804643	-0.001	0.007	0.833
cg08810410	-0.005	0.012	0.665
cg15317464	-0.001	0.014	0.956
cg25049210	0.000	0.013	0.982
cg22324029	-0.017	0.023	0.467
cg08840298	0.002	0.010	0.849
cg25953130	0.024	0.022	0.266
cg21747782	-0.002	0.009	0.853
cg09191149	0.003	0.014	0.803
cg00938688	0.006	0.012	0.600
cg09915396	-0.012	0.014	0.379
cg04492567	0.001	0.009	0.924
cg04384031	0.002	0.009	0.812
cg07408552	0.000	0.012	0.967
cg18575710	-0.001	0.010	0.960

GDM as reference group

Models were adjusted for maternal age, race and ethnicity, education attainment, smoking status during pregnancy, prepregnancy overweight or obesity, newborn’s sex, fetal growth status, and cell type composition.

Supplemental Table S4. Association between diabetes and CpG sites which were covered in significant DMRs

CpG ID	β	se	P value	Adj P	CHR	Coordinate	Target gene	Genomic location	Relation to CpG island
cg00575744	0.35	0.09	2.0E-04	0.251	6	32908642	HLA-DMB	1stExon;5'UTR	
cg10310595	0.43	0.10	7.4E-06	0.087	6	32908567	HLA-DMB	1stExon	
cg12840248	0.30	0.09	1.4E-03	0.411	6	32908718	HLA-DMB	1stExon;5'UTR	
cg16236263	0.39	0.10	5.8E-05	0.175	6	32908605	HLA-DMB	1stExon;5'UTR	
cg22324029	0.47	0.09	3.3E-07	0.031	6	32908466	HLA-DMB	Body	
cg04384031	0.34	0.06	1.0E-07	0.017	17	19631485			S_Shelf
cg04492567	0.33	0.06	2.3E-07	0.031	17	19631464			S_Shelf

Adj P, P value adjusted by multiple test correction; CHR, chromosome;

Supplemental Table S5. Differentially methylated regions identified by DMRcate in 1000 bp window

DMR Coordinates	Chr	CpGs comprising the DMR	Width	No. of CpG	Overlapping genes	Fisher	HMFDR
32908239-32909282	6	cg17022232; cg00575744 cg20392842; cg10310595 cg25247351; cg20600379 cg16300030; cg10714284 cg12840248; cg16236263 cg22324029	1044	11	HLA-DMB	0.118	0.152
11898478-11899258	20	cg20981848; cg05822633 cg18808904; cg01444716 cg04682802	781	5	BTBD3	0.140	0.176
32129623-32120783	6	cg08045906; cg12883279 cg18235088; cg12626589	161	4	PRRT1	0.984	0.766

Chr, chromosome; DMR, differentially methylated region; Fisher, Fisher's multiple comparison statistic; HMFDR, harmonic mean of the individual component FDRs.

Supplemental Table S6. Look-up analyses results

Cpg ID	coef	sd	p value	CH	Coordinate	Target Gene	Genomic location	Study	D	p_fdr
cg25049210	0.44	0.09	3.7E-07	3	30495126			Antoun	+	1.0E-04
cg25954315	0.35	0.07	3.4E-06	12	4257682			Antoun	+	4.6E-04
cg25975961	0.38	0.09	1.4E-05	7	150600818			Antoun	+	1.2E-03
cg19913563	0.33	0.08	2.8E-05	6	30720261			Antoun	+	1.9E-03
cg02430430	0.31	0.07	4.4E-05	15	74532450	CCDC33	Body	Antoun	+	2.4E-03
cg12405088	0.33	0.08	5.6E-05	22	43257120			Antoun	+	2.4E-03
cg04685228	0.35	0.09	6.3E-05	5	172462626			Antoun	+	2.4E-03
cg13245626	0.33	0.08	6.9E-05	4	75558301			Antoun	+	2.4E-03
cg14455176	-0.35	0.09	1.1E-04	1	156785998	SH2D2A; NTRK1	TSS200; Body	Antoun	-	2.9E-03
cg24129356	0.36	0.09	1.1E-04	6	32920735	HLA-DMA	1stExon	Antoun	+	2.9E-03
cg21757973	0.34	0.09	1.3E-04	10	25171393	PRTFDC1	Body	Antoun	+	2.9E-03
cg27295118	0.35	0.09	1.3E-04	14	22902226			Antoun	+	2.9E-03
cg21132686	0.36	0.09	1.4E-04	20	22795774			Antoun	+	2.9E-03
cg07338715	0.35	0.09	1.9E-04	2	109649281			Antoun	+	3.1E-03
cg08735211	0.34	0.09	1.9E-04	6	32920657	HLA-DMA	Body	Antoun	+	3.1E-03
cg11960794	0.29	0.08	2.0E-04	22	19651048			Antoun	+	3.1E-03
cg03245099	0.3	0.08	2.1E-04	1	28201862	THEMIS2	Body	Antoun	+	3.1E-03
cg12148585	0.27	0.07	2.1E-04	2	208006420	KLF7	Body	Antoun	+	3.1E-03
cg02001279	0.32	0.09	2.2E-04	19	940967	ARID3A	Body	Antoun	+	3.1E-03
cg06408559	0.32	0.09	2.9E-04	1	212963092	NSL1	Body	Antoun	+	4.0E-03
cg12856497	0.31	0.09	3.1E-04	17	55525538	MSI2	Body	Antoun	+	4.0E-03
cg12428797	0.34	0.1	3.4E-04	8	144678360	EEF1D	5'UTR	Antoun	+	4.2E-03
cg26095923	0.32	0.09	4.1E-04	10	63747939	ARID5B	Body	Antoun	+	4.9E-03
cg26195586	0.21	0.06	4.6E-04	5	138532335	SIL1	5'UTR	Antoun	+	5.2E-03
cg12758082	0.3	0.08	4.7E-04	13	113407769	ATP11A	Body	Antoun	+	5.2E-03
cg24296397	0.32	0.09	6.9E-04	3	49692537	BSN	Body	Antoun	+	7.1E-03
cg09709426	0.31	0.09	7.0E-04	3	45911521	LZTFL1	5'UTR;Body	Antoun	+	7.1E-03
cg07738730	-0.31	0.09	8.1E-04	17	47077165	IGF2BP1	Body	Antoun	-	7.9E-03
cg27518892	0.31	0.09	8.5E-04	16	57566936	CCDC102A	5'UTR	Antoun	+	8.0E-03
cg20417154	0.29	0.09	8.8E-04	12	123944398	SNRNP35	5'UTR; 1stExon;Body	Antoun	+	8.1E-03
cg09352285	0.3	0.09	9.7E-04	20	47919910			Antoun	+	8.5E-03
cg15016701	0.3	0.09	1.1E-03	12	63211683	PPM1H	Body	Antoun	+	9.0E-03
cg20409231	0.3	0.09	1.2E-03	3	156382473			Antoun	+	9.7E-03
cg11646706	0.22	0.07	1.2E-03	3	58514483	ACOX2	Body	Antoun	+	9.7E-03
cg05160449	0.28	0.09	1.3E-03	21	43580161			Antoun	+	9.7E-03
cg06233873	0.26	0.08	1.3E-03	5	137641727	CDC25C	Body	Antoun	+	9.7E-03
cg20068209	0.25	0.08	1.3E-03	6	75988568	TMEM30A	Body	Antoun	+	9.9E-03
cg03295554	0.25	0.08	1.4E-03	11	128395450	ETS1	Body	Antoun	+	1.0E-02
cg00382138	0.26	0.08	1.5E-03	4	110723299	CFI	1stExon; 5'UTR	Antoun	+	1.1E-02
cg11415687	0.3	0.1	1.7E-03	17	4806623	CHRNE	TSS1500	Antoun	+	1.1E-02
cg04347477	0.29	0.09	1.8E-03	12	125002007	NCOR2	5'UTR	Antoun	+	1.2E-02
cg25551168	0.28	0.09	2.1E-03	20	3065343	AVP	5'UTR; 1stExon	Antoun	+	1.3E-02
cg15774976	-0.29	0.09	2.1E-03	5	92953974	FAM172AA ;MIR548AO	3'UTR;Body	Antoun	-	1.3E-02
cg12822891	-0.29	0.1	2.2E-03	14	103464954	CDC42BPB	Body	Antoun	-	1.3E-02
cg18641655	0.29	0.1	2.2E-03	8	125025636	FER1L6; FER1L6-AS1	Body; Body	Antoun	+	1.3E-02
cg17133774	0.24	0.08	2.2E-03	1	6198667	CHD5	Body	Antoun	+	1.3E-02
cg26027170	0.26	0.08	2.3E-03	17	37394705			Antoun	+	1.3E-02
cg24503146	0.23	0.07	2.4E-03	17	55558110	MSI2	Body	Antoun	+	1.4E-02
cg21404980	0.25	0.08	2.8E-03	8	144599027	ZC3H3	Body	Antoun	+	1.5E-02

cg04714030	0.26	0.09	2.8E-03	10	32606973	EPC1	Body;5'UTR	Antoun	+	1.5E-02
cg25201165	0.28	0.09	2.8E-03	18	60458895	PHLPP1	Body	Antoun	+	1.5E-02
cg12713583	0.26	0.09	3.0E-03	19	940724	ARID3A	Body	Antoun	+	1.6E-02
cg00948111	0.23	0.08	3.4E-03	18	72550342	ZNF407	Body	Antoun	+	1.7E-02
cg00406098	0.24	0.08	3.4E-03	13	40716180			Antoun	+	1.7E-02
cg12592220	0.22	0.08	3.5E-03	18	42686881			Antoun	+	1.7E-02
cg06653796	0.19	0.06	3.6E-03	20	62367805	LIME1	TSS200	Antoun	+	1.7E-02
cg22692692	0.26	0.09	3.6E-03	3	194432892	LOC100507391	Body	Antoun	+	1.7E-02
cg04188920	0.24	0.08	3.9E-03	10	3138534	PFKP	Body	Antoun	+	1.8E-02
cg13687834	0.25	0.09	3.9E-03	10	3514783			Antoun	+	1.8E-02
cg20207982	0.27	0.09	4.0E-03	6	149487922			Antoun	+	1.8E-02
cg22699285	0.24	0.08	4.0E-03	12	107763727	BTBD11	Body	Antoun	+	1.8E-02
cg01824466	0.24	0.08	4.1E-03	8	95959531	TP53INP1	5'UTR	Antoun	+	1.8E-02
cg25638549	0.25	0.09	4.6E-03	10	13692225	FRMD4A	3'UTR	Antoun	+	2.0E-02
cg00717259	0.25	0.09	4.6E-03	19	39742656			Antoun	+	2.0E-02
cg19748455	0.25	0.09	4.8E-03	17	76274856	LOC100996291	TSS1500	Antoun	+	2.0E-02
cg19333758	0.24	0.09	5.8E-03	12	48135549	RAPGEF3	Body	Antoun	+	2.4E-02
cg06478995	0.24	0.09	6.0E-03	6	117003718	KPNA5	Body	Antoun	+	2.5E-02
cg11360522	0.25	0.09	6.3E-03	13	113379828	ATP11A	Body	Antoun	+	2.5E-02
cg18623216	0.2	0.07	6.4E-03	3	155421970	PLCH1	5'UTR; 1stExon	Antoun	+	2.5E-02
cg01470610	0.23	0.09	7.1E-03	13	49946235	CAB39L	Body	Antoun	+	2.7E-02
cg21180953	0.26	0.1	7.1E-03	18	42489607	SETBP1	Body	Antoun	+	2.7E-02
cg02988288	-0.23	0.09	8.0E-03	1	145440445	TXNIP; NBPF20	Body	Antoun	-	3.0E-02
cg01130991	-0.19	0.07	8.9E-03	17	46510392			Antoun	-	3.3E-02
cg00063535	-0.24	0.09	9.2E-03	12	113729491	TPCN1	Body	Haertle	+	3.4E-02
cg05493528	0.2	0.08	9.2E-03	1	65631531	AK4	Body	Antoun	+	3.4E-02
cg25715278	0.21	0.08	9.7E-03	10	126040305			Antoun	+	3.5E-02
cg24824128	0.21	0.08	9.8E-03	1	28202085	THEMIS2	Body	Antoun	+	3.5E-02
cg04668163	0.24	0.09	1.1E-02	9	133546268	PRDM12	Body	Antoun	+	3.7E-02
cg02683621	-0.23	0.09	1.1E-02	7	150100820			Haertle	+	3.9E-02
cg17747781	0.22	0.09	1.1E-02	8	143383498	TSNARE1	Body	Antoun	+	3.9E-02
cg17738521	0.17	0.06	1.2E-02	6	143224179	HIVEP2	5'UTR	Antoun	+	4.1E-02
cg09150130	0.23	0.09	1.4E-02	20	3083101			Antoun	+	4.5E-02
cg16843099	0.22	0.09	1.4E-02	5	178956830			Antoun	+	4.5E-02
cg25772839	0.22	0.09	1.5E-02	3	194573951			Antoun	+	4.9E-02
cg05960182	0.22	0.09	1.5E-02	1	16160360			Antoun	+	4.9E-02
cg02240291	-0.21	0.09	1.5E-02	7	150943825	SMARCD3	Body	Antoun	-	4.9E-02
cg20258698	0.22	0.09	1.6E-02	7	47556811	TNS3	5'UTR	Antoun	+	5.0E-02
cg21986657	0.23	0.09	1.7E-02	20	20192278	CFAP61	Body	Antoun	+	5.2E-02
cg02983090	0.21	0.09	1.7E-02	16	27437892	IL21R	TSS1500; 5'UTR	Antoun	+	5.3E-02
cg19029445	-0.23	0.1	1.7E-02	17	907732	ABR	3'UTR	Antoun	-	5.3E-02
cg10163406	0.2	0.08	1.8E-02	1	212803810			Antoun	+	5.3E-02
cg08542429	-0.2	0.09	1.8E-02	6	32139120	AGPAT1	Body	Haertle	+	5.3E-02
cg05394561	0.21	0.09	2.0E-02	17	39465682	KRTAP16-1	TSS200	Antoun	+	5.9E-02
cg03064693	0.17	0.07	2.1E-02	9	132758085	FNBP1	Body	Antoun	+	6.1E-02
cg02577793	0.21	0.09	2.2E-02	1	87174778	SH3GLB1	5'UTR;Body	Antoun	+	6.3E-02
cg08326019	-0.21	0.09	2.2E-02	19	11472261	LPPR2	Body	Antoun	-	6.3E-02
cg02669047	0.15	0.06	2.4E-02	1	206931233			Antoun	+	6.8E-02
cg00896287	0.21	0.1	2.4E-02	21	45768217			Antoun	+	6.8E-02
cg23238168	0.18	0.08	2.7E-02	17	75124189	SEC14L1	5'UTR	Antoun	+	7.5E-02
cg06583577	0.2	0.09	2.8E-02	5	115897444	SEMA6A	5'UTR	Antoun	+	7.6E-02
cg23541926	0.17	0.08	2.8E-02	3	155422092	PLCH1	TSS200	Antoun	+	7.7E-02
cg18906596	0.2	0.09	3.0E-02	17	4151473	ANKFY1	Body	Haertle	-	8.2E-02
cg18214661	0.19	0.09	3.3E-02	8	17471997	PDGFRL	Body	Antoun	+	8.7E-02
cg09827745	0.18	0.08	3.3E-02	17	72898015			Antoun	+	8.7E-02

cg02735762	0.19	0.09	3.3E-02	9	135280262	TTF1	5'UTR	Antoun	+	8.7E-02
cg01505367	-0.2	0.1	3.5E-02	15	43559094	TGM5	TSS200	Antoun	-	9.1E-02
cg03566881	0.19	0.09	3.5E-02	1	202210983	LGR6	Body	Antoun	+	9.1E-02
cg25693349	0.17	0.08	3.7E-02	6	32808596	PSMB8	3'UTR;	Antoun	+	9.4E-02
cg16536918	0.19	0.09	3.8E-02	20	3065403	AVP	TSS200	Antoun	+	9.5E-02
cg07302471	0.19	0.09	3.8E-02	3	51657172	RAD54L2	Body	Antoun	+	9.5E-02
cg04514868	-0.2	0.1	3.8E-02	14	105931040	MTA1	Body	Haertle	+	9.5E-02
cg25937862	-0.18	0.09	4.0E-02	17	60703660	MRC2	TSS1500	Antoun	-	9.9E-02
cg18347497	0.19	0.09	4.3E-02	8	60442729			Antoun	+	1.0E-01
cg02646480	-0.18	0.09	4.3E-02	19	42431565			Antoun	-	1.0E-01
cg22504849	0.14	0.07	4.4E-02	5	171385088	FBXW11	Body	Antoun	+	1.0E-01
cg25900017	-0.16	0.08	4.5E-02	11	34616427			Antoun	-	1.1E-01
cg00921839	0.18	0.09	4.6E-02	1	11467299			Antoun	+	1.1E-01
cg14868453	-0.16	0.08	4.9E-02	2	101996840	CREG2	Body	Antoun	-	1.1E-01
cg21143899	-0.19	0.09	4.9E-02	1	165866296	UCK2	Body	Haertle	+	1.1E-01
cg07624582	0.16	0.08	5.0E-02	9	14271314	NFIB	Body	Antoun	+	1.1E-01
cg19136673	0.15	0.08	5.3E-02	6	32813337	PSMB8;TAP1	TSS1500; 3'UTR	Antoun	+	1.2E-01
cg16345559	0.18	0.09	5.5E-02	2	213933386	IKZF2	Body	Antoun	+	1.2E-01
cg11932158	0.16	0.08	5.5E-02	3	155422129	PLCH1	TSS200	Antoun	+	1.2E-01
cg04632887	0.16	0.08	5.6E-02	20	3065559	AVP	TSS200	Antoun	+	1.2E-01
cg05650559	-0.19	0.1	5.8E-02	3	159201035	SCHIP1	Body	Antoun	+	1.3E-01
cg08726900	0.13	0.07	5.8E-02	16	89550474	ANKRD11	5'UTR	Antoun	+	1.3E-01
cg14295482	-0.15	0.08	5.9E-02	19	2555717	GNG7	5'UTR	Antoun	-	1.3E-01
cg03280063	-0.17	0.09	6.0E-02	4	893186	GAK	Body	Haertle	+	1.3E-01
cg13492133	-0.17	0.09	6.3E-02	1	183516266	SMG7	Body	Antoun	-	1.3E-01
cg11199014	-0.17	0.09	6.6E-02	19	39798563	LRFN1	Body	Antoun	-	1.4E-01
cg10288510	0.18	0.1	6.8E-02	1	214158727			Haertle	-	1.4E-01
cg19240637	0.12	0.06	7.0E-02	2	7172297	RNF144A	Body	Antoun	+	1.4E-01
cg08440349	-0.16	0.09	7.3E-02	16	84486704	ATP2C2	Body	Haertle	+	1.5E-01
cg15756156	-0.16	0.09	7.5E-02	22	23245724			Antoun	-	1.5E-01
cg15737302	0.16	0.09	8.2E-02	11	118302063			Haertle	-	1.7E-01
cg22805485	-0.16	0.09	8.3E-02	11	13983818	SPON1	TSS200	Antoun	-	1.7E-01
cg02306236	0.16	0.09	8.7E-02	12	123570372	PITPNM2	5'UTR	Antoun	+	1.7E-01
cg13706613	-0.16	0.09	8.7E-02	9	139324927	INPP5E	Body	Haertle	+	1.7E-01
cg17417675	0.16	0.09	9.0E-02	8	103543145			Antoun	+	1.8E-01
cg00420390	0.12	0.07	9.1E-02	7	2256023	MAD1L1	Body	Antoun	+	1.8E-01
cg26893515	0.13	0.08	9.3E-02	8	129342187			Antoun	+	1.8E-01
cg19227924	-0.15	0.09	9.4E-02	17	28565709			Antoun	-	1.8E-01
cg05351447	0.12	0.07	9.5E-02	10	119120604	PDZD8	Body	Antoun	+	1.8E-01
cg00237080	-0.15	0.09	9.8E-02	19	46899291			Antoun	-	1.9E-01
cg21086113	0.15	0.09	9.8E-02	16	27760233	KIAA0556	Body	Antoun	+	1.9E-01
cg21262198	0.14	0.09	1.0E-01	3	155422159	PLCH1	TSS200	Antoun	+	2.0E-01
cg26077811	-0.13	0.08	1.1E-01	11	119232263	USP2	Body	Antoun	-	2.0E-01
cg12841566	-0.14	0.09	1.1E-01	11	47296317	MADD	Body	Haertle	+	2.0E-01
cg02609480	0.12	0.07	1.1E-01	21	46971551			Antoun	+	2.1E-01
cg04350530	0.14	0.09	1.1E-01	4	140728329	MAML3	Body	Antoun	+	2.1E-01
cg25676030	0.1	0.07	1.2E-01	3	4946693	BHLHE40-AS1	Body	Antoun	+	2.1E-01
cg03425812	0.08	0.05	1.2E-01	15	45005363	B2M	Body	Antoun	+	2.2E-01
cg11449134	-0.15	0.1	1.2E-01	19	51897791			Haertle	-	2.2E-01
cg24769355	0.14	0.09	1.2E-01	9	74656792			Antoun	+	2.2E-01
cg20393308	-0.13	0.09	1.4E-01	17	43728044	MGC57346-CRHR1	5'UTR	Antoun	-	2.5E-01
cg15877233	-0.12	0.08	1.5E-01	7	40611688	C7orf10	Body	Antoun	-	2.6E-01
cg09673812	0.14	0.09	1.5E-01	19	30469225	URI1	Body	Antoun	+	2.6E-01
cg24914185	0.12	0.08	1.5E-01	2	200663789			Antoun	+	2.6E-01
cg03345925	0.11	0.08	1.5E-01	8	144599347	ZC3H3	Body	Haertle	+	2.6E-01
cg27109588	-0.09	0.07	1.5E-01	3	128930686			Antoun	+	2.6E-01
cg12595459	0.11	0.08	1.6E-01	8	28527302			Antoun	+	2.7E-01
cg03036214	0.12	0.09	1.6E-01	15	63640658	CA12	Body	Antoun	+	2.7E-01
cg05077231	-0.11	0.08	1.7E-01	13	49793054	MLNR	TSS1500	Antoun	-	2.8E-01

cg07689396	0.09	0.07	1.7E-01	7	633050	PRKAR1B	Body	Haertle	+	2.8E-01
cg00327947	0.11	0.08	1.7E-01	16	85482849			Antoun	+	2.8E-01
cg04174091	0.08	0.06	1.7E-01	11	122935222			Antoun	+	2.8E-01
cg19617213	0.11	0.08	1.7E-01	19	1074926	HMHA1	Body	Antoun	+	2.8E-01
cg08144943	-0.13	0.09	1.8E-01	3	52280702	PPM1M	5'UTR	Haertle	+	2.9E-01
cg16326902	-0.11	0.08	1.8E-01	20	49253364	FAM65C	1stExon; 5'UTR	Antoun	-	2.9E-01
cg04354433	0.12	0.09	1.8E-01	6	32256343			Antoun	+	3.0E-01
cg04345034	0.11	0.09	1.9E-01	15	70797389			Antoun	+	3.0E-01
cg25156118	-0.12	0.09	1.9E-01	19	39799137	LRFN1	Body	Antoun	-	3.1E-01
cg18502630	-0.12	0.09	2.0E-01	9	139871955	PTGDS	5'UTR; 1stExon	Haertle	+	3.1E-01
cg26001655	-0.12	0.09	2.0E-01	4	1356770	KIAA1530	Body	Haertle	+	3.1E-01
cg25592832	0.12	0.09	2.0E-01	19	55009051			Antoun	+	3.1E-01
cg17881203	-0.12	0.1	2.0E-01	19	990398	WDR18	Body	Haertle	+	3.1E-01
cg07431064	0.12	0.09	2.0E-01	22	39529217	CBX7	3'UTR	Haertle	+	3.1E-01
cg00506306	0.11	0.09	2.0E-01	7	51207974	COBL	Body	Antoun	+	3.2E-01
cg22523078	-0.12	0.09	2.1E-01	15	74417907			Antoun	-	3.3E-01
cg20734092	0.11	0.09	2.2E-01	10	22546132	LOC100130 992	Body	Antoun	+	3.3E-01
cg01205011	0.11	0.09	2.3E-01	6	35262113	ZNF76	Body	Haertle	-	3.4E-01
cg13153307	-0.12	0.1	2.3E-01	9	139368749	SEC16A	Body	Haertle	+	3.4E-01
cg18679416	0.08	0.06	2.3E-01	2	158261028			Antoun	+	3.4E-01
cg03814573	0.08	0.07	2.3E-01	12	120691486	PXN	Body	Antoun	+	3.5E-01
cg03186149	0.11	0.09	2.3E-01	19	46877200	PPP5C	Body	Antoun	+	3.5E-01
cg22163280	-0.11	0.09	2.4E-01	17	72423543			Antoun	-	3.5E-01
cg13741394	-0.11	0.09	2.4E-01	18	43417389	SIGLEC15	Body	Antoun	-	3.5E-01
cg27478579	0.11	0.09	2.4E-01	2	55497012	MTIF2	TSS1500	Antoun	+	3.5E-01
cg13932501	0.06	0.05	2.5E-01	9	94060853	AUH	Body	Antoun	+	3.6E-01
cg12533384	0.09	0.08	2.5E-01	6	130182155	TMEM244	Body	Antoun	+	3.7E-01
cg01601105	0.06	0.06	2.6E-01	1	16173292	SPEN; FLJ37453	TSS1500; Body	Antoun	+	3.7E-01
cg05669550	-0.08	0.07	2.6E-01	15	101728451	CHSY1	Body	Antoun	-	3.7E-01
cg23007418	0.06	0.05	2.6E-01	17	76731885	CYTH1	Body	Antoun	+	3.7E-01
cg19777352	0.1	0.09	2.8E-01	11	47082713	C11orf49	Body	Antoun	+	3.9E-01
cg11666770	-0.09	0.08	2.8E-01	19	42430910			Antoun	-	4.0E-01
cg14620184	-0.09	0.09	2.9E-01	2	29941470	ALK	Body	Antoun	-	4.1E-01
cg21131729	-0.1	0.09	3.1E-01	20	555298			Antoun	-	4.3E-01
cg06396119	-0.08	0.08	3.2E-01	13	49792767			Antoun	-	4.4E-01
cg10394757	-0.08	0.08	3.2E-01	16	66879807	CA7	Body;5'U TR	Antoun	-	4.4E-01
cg04037585	-0.09	0.09	3.2E-01	16	56231292	GNAO1	Body	Antoun	-	4.4E-01
cg20858400	-0.09	0.09	3.3E-01	14	104928652			Antoun	+	4.4E-01
cg02943336	-0.09	0.09	3.3E-01	7	2959067	CARD11	Body	Haertle	+	4.4E-01
cg18317026	-0.09	0.1	3.3E-01	15	54852175	UNC13C	Body	Antoun	+	4.4E-01
cg18728406	-0.07	0.07	3.3E-01	12	50066533	FMNL3	Body	Antoun	-	4.4E-01
cg07805345	-0.07	0.08	3.3E-01	3	46922486	PTH1R	5'UTR	Antoun	-	4.4E-01
cg08443019	-0.09	0.09	3.3E-01	17	1946299	OVCA2; DPH1	Body; 3'UTR	Haertle	+	4.4E-01
cg22907232	0.09	0.09	3.4E-01	3	12486268			Antoun	-	4.5E-01
cg01704198	0.07	0.08	3.5E-01	3	33757893	CLASP2	Body	Antoun	+	4.6E-01
cg16126178	-0.09	0.09	3.6E-01	14	105239857	AKT1	Body	Haertle	+	4.7E-01
cg04286826	0.05	0.06	3.6E-01	2	99386505	LOC101927 070	Body	Antoun	+	4.7E-01
cg10726445	0.06	0.06	3.6E-01	13	76336809	LMO7	5'UTR;Bo dy	Antoun	+	4.7E-01
cg00166343	0.08	0.09	3.6E-01	17	29150100	CRLF3	Body	Antoun	+	4.7E-01
cg22606873	0.08	0.09	3.7E-01	1	3144679	PRDM16	Body	Haertle	-	4.7E-01
cg01798157	0.06	0.07	3.7E-01	1	203276595	BTG2	3'UTR	Antoun	+	4.7E-01
cg26828643	-0.08	0.09	3.7E-01	16	88802820	FAM38A	Body	Haertle	+	4.7E-01
cg08450478	0.08	0.09	3.7E-01	1	78955823	PTGFR	TSS1500	Antoun	+	4.7E-01
cg19169154	0.08	0.09	3.9E-01	17	19287978	MFAP4	Body	Haertle	+	4.9E-01

cg18765405	0.08	0.09	3.9E-01	5	134644263			Antoun	+	4.9E-01
cg16063640	-0.08	0.09	4.0E-01	10	102807511			Antoun	-	4.9E-01
cg19750657	-0.07	0.08	4.0E-01	13	38935967	UFM1	3'UTR	Antoun	-	4.9E-01
cg12226023	-0.07	0.09	4.1E-01	2	219747079	WNT10A	Body	Antoun	-	5.1E-01
cg24873093	0.08	0.09	4.2E-01	1	45991060			Antoun	+	5.2E-01
cg22451300	0.05	0.06	4.3E-01	1	61692418	NFIA	Body	Antoun	+	5.3E-01
cg10778517	-0.07	0.09	4.4E-01	7	2252773	MAD1L1	Body	Haertle	+	5.4E-01
cg19143209	-0.07	0.1	4.5E-01	9	19789287			Haertle	-	5.4E-01
cg13733403	-0.07	0.1	4.6E-01	19	6528291			Antoun	-	5.5E-01
cg26910511	-0.07	0.1	4.6E-01	19	39799037	LRFN1	Body	Antoun	-	5.5E-01
cg00273340	-0.07	0.1	4.7E-01	11	64112444	CCDC88B	Body	Haertle	+	5.7E-01
cg19736654	-0.07	0.09	4.7E-01	19	6746221	TRIP10	Body	Antoun	-	5.7E-01
cg03750061	0.06	0.08	4.8E-01	10	119131167	PDZD8	Body	Antoun	+	5.7E-01
cg14597908	0.06	0.09	5.2E-01	20	57414960	GNASAS; GNAS	Body; 1stExon; 5'UTR	Haertle	+	6.1E-01
cg01893462	0.05	0.08	5.2E-01	7	139423617	HIPK2	Body	Antoun	+	6.1E-01
cg09244071	-0.06	0.09	5.2E-01	7	101768746	CUX1	Body	Haertle	+	6.1E-01
cg17372327	-0.06	0.09	5.3E-01	17	57503086			Antoun	-	6.2E-01
cg14321492	0.04	0.07	5.4E-01	8	68598797	CPA6	Body	Antoun	+	6.3E-01
cg07283598	-0.06	0.09	5.4E-01	2	236015654			Antoun	-	6.3E-01
cg27184903	-0.06	0.09	5.4E-01	15	29285727	APBA2	5'UTR	Antoun	-	6.3E-01
cg08732684	-0.05	0.08	5.6E-01	6	32095128	ATF6B	Body	Haertle	+	6.5E-01
cg08077807	0.05	0.09	5.6E-01	14	62001072	PRKCH	Body	Haertle	-	6.5E-01
cg07018980	-0.05	0.09	5.8E-01	4	895604	GAK	Body	Haertle	+	6.6E-01
cg05536286	-0.05	0.09	6.0E-01	15	92972514	ST8SIA2	Body	Haertle	-	6.8E-01
cg17945422	0.05	0.09	6.1E-01	18	76483175			Antoun	+	6.9E-01
cg19830000	0.05	0.1	6.2E-01	12	45270312	NELL2	Body; TSS1500; TSS200	Haertle	-	7.0E-01
cg14681242	-0.04	0.09	6.2E-01	17	47651550	LOC100288 866	TSS200	Antoun	-	7.0E-01
cg14088574	0.04	0.09	6.4E-01	6	33234976	VPS52	Body	Haertle	+	7.1E-01
cg09361367	0.05	0.1	6.5E-01	10	53462824	PRKG1	Body	Antoun	+	7.2E-01
cg22865713	0.03	0.08	6.7E-01	6	25779897	SLC17A4	3'UTR	Haertle	-	7.5E-01
cg25927444	0.04	0.1	6.9E-01	2	47236103	TTC7A	Body	Haertle	-	7.6E-01
cg10573018	0.04	0.1	6.9E-01	1	38100837	RSPO1	TSS1500	Antoun	-	7.6E-01
cg07377446	-0.03	0.09	7.1E-01	19	39754498			Antoun	-	7.8E-01
cg23925513	-0.03	0.09	7.2E-01	17	6923526			Antoun	-	7.9E-01
cg01993865	-0.03	0.1	7.3E-01	20	17550690	DSTN	5'UTR; 1stExon	Haertle	-	7.9E-01
cg01408926	0.03	0.07	7.3E-01	19	49907512	CCDC155	Body	Antoun	-	7.9E-01
cg17427926	-0.02	0.07	7.4E-01	17	76227386	LOC283999	TSS200	Antoun	-	7.9E-01
cg01968402	-0.03	0.1	7.5E-01	6	137817775			Haertle	+	8.0E-01
cg01955962	0.03	0.1	7.7E-01	15	73089536			Haertle	-	8.2E-01
cg20935025	-0.02	0.09	8.2E-01	14	35874013	NFKBIA	TSS200	Haertle	+	8.7E-01
cg17921080	0.02	0.08	8.2E-01	14	86478932			Haertle	-	8.7E-01
cg10576992	0.02	0.09	8.2E-01	4	119662530	SEC24D	Body	Haertle	-	8.7E-01
cg15475323	-0.02	0.09	8.3E-01	19	49220102	MAMSTR	1stExon; Body;5'U TR	Antoun	-	8.8E-01
cg00730857	-0.02	0.09	8.5E-01	4	1994281	WHSC2	Body	Haertle	+	8.9E-01
cg10248470	-0.02	0.1	8.5E-01	6	33790551			Antoun	-	8.9E-01
cg00872144	-0.02	0.1	8.7E-01	20	61626187			Antoun	-	9.1E-01
cg01203331	0.01	0.09	8.8E-01	20	2636597	SNORD57; NOP56; SNORD86	TSS1500; Body; TSS200	Haertle	+	9.1E-01
cg27455061	-0.01	0.1	8.8E-01	2	144498168	ARHGAP15	Body	Antoun	+	9.1E-01
cg04078644	0.01	0.07	8.9E-01	3	155458975			Haertle	-	9.1E-01
cg05697697	0.01	0.1	8.9E-01	10	111683345	XPNPEP1	TSS200	Haertle	+	9.2E-01
cg27005487	0.01	0.09	9.1E-01	17	59484388	TBX2	Body	Antoun	-	9.3E-01
cg26281025	0.01	0.09	9.3E-01	5	176308046	HK3	3'UTR	Haertle	+	9.5E-01

cg23376861	-0.01	0.07	9.4E-01	18	43678713	ATP5A1	5'UTR; TSS1500	Haertle	-	9.5E-01
cg20563452	0.01	0.1	9.6E-01	8	1424554			Antoun	+	9.7E-01
cg11703745	0	0.09	9.7E-01	1	205199293	TMCC2	Body	Haertle	+	9.8E-01
cg14689317	0	0.08	9.8E-01	11	64128973	RPS6KA4	Body	Antoun	-	9.8E-01
cg19193422	0	0.09	1.0E+00	11	131942222	NTM	Body	Antoun	-	1.0E+00

Abbreviations: CH, chromosome; Coef, coefficient; D, direction; +, positive; -, negative

Supplemental Table S7. Top 10 enriched pathways in KEGG for differentially methylated probes

ID	KEGG pathway	N	DE	P value
path:hsa05323	Rheumatoid arthritis	86	2	0.0011
path:hsa04721	Synaptic vesicle cycle	78	2	0.0030
path:hsa04145	Phagosome	137	2	0.0039
path:hsa05152	Tuberculosis	162	2	0.0060
path:hsa05310	Asthma	23	1	0.0073
path:hsa05330	Allograft rejection	30	1	0.0113
path:hsa05332	Graft-versus-host disease	34	1	0.0120
path:hsa05320	Autoimmune thyroid disease	39	1	0.0173
path:hsa04672	Intestinal immune network for IgA production	41	1	0.0182
path:hsa04966	Collecting duct acid secretion	27	1	0.0189

KEGG, Kyoto Encyclopedia of Genes and Genomes; ID, pathway identifier; N, number of genes in pathway; DE, number of genes that are differentially methylated; p value for over-representation of the KEGG pathway.

Supplemental Table S8. Top 10 enriched terms in Gene-Ontology (GO) molecular function, biological processes, and cell compartment identified for differentially methylated probes

ID	ONT	GO term	N	DE	P value
GO:0010121	BP	arginine catabolic process to proline via ornithine	1	1	0.0006
GO:0019493	BP	arginine catabolic process to proline	1	1	0.0006
GO:0019544	BP	arginine catabolic process to glutamate	1	1	0.0006
GO:0006561	BP	proline biosynthetic process	6	1	0.0022
GO:0004587	MF	ornithine-oxo-acid transaminase activity	1	1	0.0006
GO:0050155	MF	ornithine(lysine) transaminase activity	1	1	0.0006
GO:0031798	MF	type 1 metabotropic glutamate receptor binding	1	1	0.0021
GO:0061828	CC	apical tubulobulbar complex	1	1	0.0021
GO:0061829	CC	basal tubulobulbar complex	1	1	0.0021
GO:0098844	CC	postsynaptic endocytic zone membrane	1	1	0.0021

GO, Gene Ontology; ONT, Ontology; BP, biological process; CC, cell compartment; MF, molecular function; ID, GO identifier; N, number of genes in the GO term; DE, number of genes that are differentially methylated; p value for over-representation of the GO term.

Supplemental Table S9. Top 10 enriched pathways in KEGG for differentially methylated region

ID	KEGG pathway	N	DE	P value
**DMR in 500 bp window**				
path:hsa05310	Asthma	23	1	0.0015
path:hsa05330	Allograft rejection	30	1	0.0027
path:hsa05332	Graft-versus-host disease	34	1	0.0027
path:hsa05320	Autoimmune thyroid disease	39	1	0.0037
path:hsa04672	Intestinal immune network for IgA production	41	1	0.0048
path:hsa05150	Staphylococcus aureus infection	85	1	0.0051
path:hsa04940	Type I diabetes mellitus	37	1	0.0053
path:hsa05322	Systemic lupus erythematosus	112	1	0.0062
path:hsa04612	Antigen processing and presentation	63	1	0.0090
path:hsa05321	Inflammatory bowel disease	58	1	0.0106
**DMR in 1000 bp window**				
path:hsa05310	Asthma	23	1	0.0015
path:hsa05330	Allograft rejection	30	1	0.0025
path:hsa05332	Graft-versus-host disease	34	1	0.0025
path:hsa05320	Autoimmune thyroid disease	39	1	0.0037
path:hsa04672	Intestinal immune network for IgA production	41	1	0.0045
path:hsa05150	Staphylococcus aureus infection	85	1	0.0050
path:hsa04940	Type I diabetes mellitus	37	1	0.0052
path:hsa05322	Systemic lupus erythematosus	112	1	0.0063
path:hsa04612	Antigen processing and presentation	63	1	0.0088
path:hsa05321	Inflammatory bowel disease	58	1	0.0104

DMR, differentially methylated region; KEGG, Kyoto Encyclopedia of Genes and Genomes; ID, pathway identifier; N, number of genes in pathway; DE, number of genes that are differentially methylated; p value for over-representation of the KEGG pathway.

Supplemental Table S10. Top 10 enriched terms in GO (Gene Ontology) molecular function, biological processes, and cell compartment identified for differentially methylated region

ID	ONT	GO term	N	DE	P value
**DMRs in 500 bp window**					
**GO:2001190**	BP	positive regulation of T cell activation via T cell receptor contact with antigen bound to MHC molecule on antigen presenting cell	3	1	0.0008
**GO:2001188**	BP	regulation of T cell activation via T cell receptor contact with antigen bound to MHC molecule on antigen presenting cell	6	1	0.0009
**GO:0016240**	BP	autophagosome membrane docking	7	1	0.0016
**GO:1901896**	BP	positive regulation of ATPase-coupled calcium transmembrane transporter activity	4	1	0.0017
**GO:0002399**	BP	MHC class II protein complex assembly	11	1	0.0019
**GO:0002503**	BP	peptide antigen assembly with MHC class II protein complex	11	1	0.0019
**GO:0002291**	BP	T cell activation via T cell receptor contact with antigen bound to MHC molecule on antigen presenting cell	10	1	0.0019
**GO:0002501**	BP	peptide antigen assembly with MHC protein complex	13	1	0.0020
**GO:1901894**	BP	regulation of ATPase-coupled calcium transmembrane transporter activity	10	1	0.0023
**GO:0042613**	CC	MHC class II protein complex	12	1	0.0019
**DMRs in 1000 bp window**					
**GO:2001190**	BP	positive regulation of T cell activation via T cell receptor contact with antigen bound to MHC molecule on antigen presenting cell	3	1	0.0009
**GO:2001188**	BP	regulation of T cell activation via T cell receptor contact with antigen bound to MHC molecule on antigen presenting cell	6	1	0.0010
**GO:0002399**	BP	MHC class II protein complex assembly	11	1	0.0018
**GO:0002503**	BP	peptide antigen assembly with MHC class II protein complex	11	1	0.0018
**GO:0002501**	BP	peptide antigen assembly with MHC protein complex	13	1	0.0019
**GO:0002291**	BP	T cell activation via T cell receptor contact with antigen bound to MHC molecule on antigen presenting cell	10	1	0.0020
**GO:0002396**	BP	MHC protein complex assembly	14	1	0.0024
**GO:0019886**	BP	antigen processing and presentation of exogenous peptide antigen via MHC class II	24	1	0.0033
**GO:0042613**	CC	MHC class II protein complex	12	1	0.0018
**GO:0042611**	CC	MHC protein complex	19	1	0.0026

DMR, differentially methylated region; GO, Gene Ontology; ONT, Ontology; BP, biological process; CC, cell compartment; MF, molecular function; ID, GO identifier; N, number of genes in the GO term; DE, number of genes that are differentially methylated; p value for over-representation of the GO term.

Supplemental Table S11. Association of diabetes in pregnancy and differentially methylated positions (DMPs) with preterm birth

	Overall preterm			Spontaneous preterm			Medically indicated preterm		
**Predictor**	OR(95%CI)	P value	Adj.P	OR(95%CI)	P value	Adj.P	OR(95%CI)	P value	Adj.P
Diabetes	2.60(1.43, 4.68)	0.002	-	1.90(0.95, 3.70)	0.061	-	4.16(1.66, 10.18)	0.002	-
cg19032863	0.88(0.7,1.09)	2.4E-01	2.4E-01	0.82(0.64,1.04)	1.1E-01	1.1E-01	1.01(0.68,1.5)	9.7E-01	9.7E-01
cg18997837	3.08(2.29,4.22)	6.5E-13	2.9E-12	2.92(2.11,4.12)	2.9E-10	1.3E-09	4.58(2.58,8.63)	7.0E-07	2.7E-06
cg21363811	0.52(0.4,0.67)	4.2E-07	5.0E-07	0.55(0.42,0.72)	2.5E-05	3.0E-05	0.38(0.23,0.59)	4.8E-05	7.8E-05
cg24804643	1.77(1.39,2.26)	4.6E-06	5.2E-06	1.63(1.25,2.14)	4.0E-04	4.2E-04	2.33(1.51,3.71)	2.1E-04	2.6E-04
cg08810410	2.57(1.95,3.44)	7.8E-11	2.3E-10	2.35(1.73,3.22)	6.6E-08	1.5E-07	3.73(2.22,6.54)	1.7E-06	4.3E-06
cg15317464	1.99(1.56,2.57)	5.2E-08	7.2E-08	1.87(1.43,2.45)	4.9E-06	6.8E-06	2.51(1.61,4.04)	8.5E-05	1.3E-04
cg25049210	3.66(2.66,5.14)	1.0E-14	1.4E-13	3.41(2.43,4.93)	1.0E-11	9.0E-11	5.86(3.12,11.89)	1.9E-07	1.9E-06
cg22324029	1.87(1.47,2.4)	4.2E-07	5.0E-07	1.8(1.39,2.36)	1.3E-05	1.6E-05	2.13(1.38,3.38)	8.7E-04	9.7E-04
cg08840298	2.33(1.79,3.05)	4.5E-10	8.1E-10	2.19(1.65,2.95)	1.1E-07	1.7E-07	3.34(2.06,5.66)	2.6E-06	5.8E-06
cg25953130	2.55(1.9,3.46)	1.1E-09	1.6E-09	2.51(1.81,3.54)	6.6E-08	1.5E-07	2.51(1.53,4.26)	4.0E-04	4.8E-04
cg21747782	2.64(2.02,3.5)	3.3E-12	1.2E-11	2.44(1.83,3.32)	4.1E-09	1.5E-08	4.1(2.47,7.21)	2.1E-07	1.9E-06
cg09191149	2.37(1.81,3.14)	7.9E-10	1.3E-09	2.33(1.73,3.19)	4.7E-08	1.4E-07	2.67(1.65,4.46)	1.1E-04	1.5E-04
cg00938688	2.42(1.85,3.22)	4.1E-10	8.1E-10	2.23(1.67,3.03)	1.2E-07	1.7E-07	3.45(2.08,6.02)	4.4E-06	7.9E-06
cg09915396	3.44(2.53,4.78)	2.5E-14	1.5E-13	3.42(2.43,4.92)	7.3E-12	9.0E-11	4.32(2.48,7.83)	5.5E-07	2.7E-06
cg04492567	2.42(1.86,3.19)	1.6E-10	4.0E-10	2.25(1.68,3.06)	9.0E-08	1.6E-07	3.77(2.24,6.68)	1.7E-06	4.3E-06
cg04384031	2.41(1.85,3.18)	2.1E-10	4.7E-10	2.25(1.68,3.06)	8.8E-08	1.6E-07	3.41(2.07,5.89)	3.8E-06	7.6E-06
cg07408552	3.65(2.65,5.13)	1.5E-14	1.4E-13	3.42(2.42,4.95)	1.5E-11	9.0E-11	4.97(2.71,9.74)	7.5E-07	2.7E-06
cg18575710	1.66(1.32,2.11)	2.1E-05	2.2E-05	1.6(1.24,2.07)	2.8E-04	3.2E-04	1.92(1.26,2.95)	2.6E-03	2.7E-03

Abbreviations: Adj P, P value adjusted by multiple test correction; OR, odds ratio.
Models were adjusted for maternal age, race and ethnicity, education attainment, smoking status during pregnancy, prepregnancy overweight or obesity, newborn’s sex, fetal growth status, and cell type composition.

Supplemental Table S12. Identification of potential mediators and covariates

Potential mediator/confounder or covariate	P-Value 1	P-Value 2
cg19032863	0.333	NA
cg18997837	0.292	NA
cg21363811	0.608	NA
cg24804643	0.165	NA
cg08810410 *	0.041	0.000
cg15317464	0.528	NA
cg25049210	0.620	NA
cg22324029	0.219	NA
cg08840298	0.302	NA
cg25953130 *	0.008	0.000
cg21747782	0.705	NA
cg09191149 *	0.004	0.000
cg00938688	0.487	NA
cg09915396 *	0.016	0.000
cg04492567	0.844	NA
cg04384031	0.868	NA
cg07408552 *	0.000	0.000
cg18575710	0.821	NA
Maternal age at delivery *	0.080	0.000
Maternal education	0.424	NA
Smoking during pregnancy	0.208	NA
Race/ethnicity	0.952	NA
Maternal overweight or obesity at pregnancy	0.830	NA
Neonatal sex	0.259	NA
Neonatal birthweight -	0.000	0.241

Note: P-value 1 is the p-value of Type III test for the row variable in the full model. P-value 2 shows the p-value of testing the association between diabetes during pregnancy and the row variables.

* indicates that the variable was chosen as a mediator/confounder, - indicates that the variable was chosen as a covariate.

Supplemental Table S13. Mediation effect of CpGs on the association of diabetes in pregnancy with medically indicated preterm birth

Mediator	TE/DE (95%CI)	IE (95%CI)	RE (%)
Total effect	2.383(1.141, 4.318)		
Direct effect	0.527(-0.433, 1.686)		0.221(-0.288, 3.407)
cg08810410		0.016(-0.385, 0.449)	-0.007(-0.471, 0.484
cg25049210*		0.851(0.229, 1.644)	0.357(-0.364, 1.062)
cg25953130		0.131(-0.144, 0.450)	0.055(-0.150, 0.265)
cg09191149		0.163(-0.104, 0.496)	0.068(-0.116, 0.267)
cg09915396		0.177(-0.081, 0.486)	0.074(-0.123, 0.271)
cg07408552		0.223(-0.006, 0.623)	0.094(-0.746, 0.955)
Joint effect of CpGs		1.497(0.924, 2.741)	0.628(-1.511, 2.846)

DE, direct effect; IE, indirect effect; RE, relative effect; TE, total effect.

Supplemental Table S14. Comparison of characteristics between total BBC and sample included in this study

Characteristics	Sample in total BBC	Sample included in this study	P value
n	8623	954	
**Maternal characteristics**			
Age (years)	28.2 (6.5)	28.3 (6.6)	0.480
Race/ethnicity (Black)	4087 (47.4)	679 (71.2)	<0.001
Parity (multiparous)	4892 (56.7)	526 (55.1)	0.363
Smoker during pregnancy	1700 (19.7)	165 (17.3)	0.081
Education (College and above)	3034 (35.2)	320 (33.5)	0.331
Overweight or obesity	4161 (46.3)	506 (53.0)	0.096
Preexisting/gestational diabetes			0.628
No	7498 (87.0)	828 (86.8)	
Yes	1117 (13.0)	126 (13.2)	
NA	8 (0.1)	0 (0.0)	
**Newborn's characteristics**			
sex (male)	4296 (49.8)	504 (52.8)	0.084
Low birthweight	2268 (26.3)	158 (16.6)	<0.001
Preterm	2364 (27.4)	173 (18.1)	<0.001
Fetal growth			0.128
AGA	6735(78.1)	754 (79.0)	
SGA	1052 (12.2)	97 (10.2)	
LGA	836 (9.7)	103 (10.8)	

AGA, appropriate for gestational age; BBC, Boston Birth Cohort; LGA, large for gestational age; SGA, small for gestational age
